# Multiple surgeries in paediatric patients with myelomeningocele. Can we define quantitative and qualitative evolution patterns according to the level of spinal lesion?

**DOI:** 10.1186/1743-8454-7-S1-S15

**Published:** 2010-12-15

**Authors:** Eulália Calado, Humberto Marreiros, Clara Loff

**Affiliations:** 1 Paediatric Neurology Department, Hospital Dona Estefânia, Lisboa, 1169-045, Portugal; 2 Paediatric Rehabilitation Department, Hospital Dona Estefânia, Lisboa, 1169-045, Portugal; 3 Paediatric Rehabilitation Department, Hospital Dona Estefânia, Lisboa, 1169-045, Portugal

## Background

Children with myelomeningocele are usually submitted to multiple surgeries, since first days of life to late adolescence, with long stays in hospital and family life disruption. However the number and type of surgeries are not the same in every child with myelomeningocele across the time and from child to child. We have attempted to define a pattern of surgeries in patients with myelomeningocele and Chiari II malformation across the time (0-18 years), according to the spinal level.

## Materials and methods

Data of all patients with myelomeningocele, followed actually in our centre, were analysed retrospectively. The inclusion criteria were: child with both myelomeningocele and Chiari II malformation, aged 0-18 years, alive and observed during 2009. The factors analysed were: date of birth, sex, spinal level, Chiari II malformations with and without shunt and the number and type of surgical interventions. The spinal level was classified in thoracic, upper lumbar level, lower lumbar level and sacral using “The International Myelodysplasia Study Group” criteria for assigning motor level. Three age groups were considered: 0-5 years; 6-12years; 13-18years. We analysed the associations across the time between the frequency of each type of surgical intervention (neurosurgical, orthopaedic, urinary and ulcers repair) and the level of motor involvement. Statistical analysis was done using the statistical package SPSS.

## Results

From a total of 153 patients with neural tube defects, 72 fulfilled the inclusion criteria, 57 with shunt (all but one ventriculoperitoneal) and 15 with arrested hydrocephalus. The median of ages was 10 years. Neurosurgical interventions predominated in the first five years of life, mostly in patients with shunts, with no relation to cord level. Orthopaedic surgeries were more frequent in the second age group (6-12 years) and in those with upper lumbar level lesions. Urological surgeries were done mainly in the 6-12 years group with lower lumbar level impairment. Surgical repair of ulcers were more frequent in ambulant adolescents (lower lumbar and sacral levels).

## Conclusions

The construction of an evolution pattern of the type and frequency of surgeries across lifespan of an important subgroup of children born with Spina Bifida and its relation with the spinal level, can be challenging for the comparison of Spina Bifida populations from different centres. Methodologies of treatment and results could be better compared among centres by benchmarking of best practices.

